# Immune priming reshapes the microbiota and modulates pathogen dynamics in the Manila clam (*Ruditapes philippinarum*)

**DOI:** 10.3389/fmicb.2026.1803024

**Published:** 2026-04-10

**Authors:** Bruno K. Rodino-Janeiro, Diego Rey-Varela, Ana L. Dieguez, Sergio Rodriguez, Clara Martinez, Javier Dubert

**Affiliations:** 1 Instituto de Investigación Sanitaria de Santiago de Compostela (IDIS), Santiago de Compostela, A Coruña, Spain; 2 Department of Microbiology and Parasitology, Aquatic One Health Research Center (ARCUS), CIBUS bldg—Faculty of Biology, University of Santiago de Compostela, Santiago de Compostela, Spain

**Keywords:** bivalve aquaculture, immune priming, microbiota shifts, pathogen dynamics, *Ruditapes philippinarum*, *Vibrio europaeus*, vibriosis

## Abstract

Invertebrates lack adaptive immunity and rely exclusively on innate defenses to combat pathogens. Recent studies have shown that invertebrates exhibit immune priming, a form of innate immune memory in which prior pathogen exposure enhances protection against reinfections. Despite its ecological and applied relevance, immune priming remains poorly explored in marine invertebrates. Most studies adopt a host-centric view, focusing on the immune response while overlooking that immunity occurs within a complex microbial context in which the host, its microbiota, and pathogens interact. This gap limits our understanding of how microbiota and pathogens behave under priming pressure. Here, we investigated the effects of immune priming on host survival, pathogen dynamics, and microbiota composition in the Manila clam (*Ruditapes philippinarum*), a key species for global aquaculture, challenged with the bacterial pathogen *Vibrio europaeus*. Immune priming was induced orally using live *V. europaeus* at a sublethal dose, followed by a lethal secondary challenge. Primed clams exhibited a significant increase in survival after the second challenge (87% survival vs. 0% in non-primed clams) and rapidly reduced the pathogen concentration within 48 h post-challenge below the mortality threshold observed in non-primed clams (~10^5^ copies mg^−1^). Notably, the pathogen was detected at low but non-harmful concentrations (~10^2^ copies mg^−1^) in primed clams during both challenges. Full-length 16S rRNA amplicon sequencing analyses revealed that immune priming reshaped the host microbiota. Beta diversity analyses suggest the establishment of a specific bacterial community in primed clams. Clustering analyses identified a priming-associated microbiota dominated by *Acinetobacter, Brevundimonas, Sphingobium*, and *Psychrobacter*, which persisted at the end of the priming and the secondary challenge but was absent or depleted in non-primed clams. In contrast, members of *Arcobacteraceae* emerged only during the second challenge, decreasing in primed clams but increasing in non-primed clams in association with high mortality. This study provides the first phenotypic evidence of oral immune priming in the Manila clam, identifies a specific microbiota and detects the pathogen despite effective host protection. This offers a new perspective to address immune priming as an emergent property of dynamic interactions between the host, its microbiota, and the pathogen.

## 1 Introduction

For decades, immunological memory was considered an exclusive trait for vertebrates, tightly linked to the adaptive immune system and mediated by antigen-specific responses involving somatic rearrangement and clonal expansion of lymphocytes (Sompayrac, 2022). This system enables vertebrates to acquire long-lasting immunity against emerging pathogens and to rapidly adapt to new immunological challenges. However, over the past two decades, accumulating evidence has demonstrated that invertebrates also possess an antigen-independent form of immunological memory (Pradeu and Du Pasquier, 2018). This phenomenon—referred to as innate immune memory, trained immunity, or immune priming—confers enhanced protection upon secondary exposure to pathogens despite the absence of adaptive immunity (Low and Chong, 2020; Montagnani et al., 2024). Immune priming elicits a vaccination-like effect, whereby prior exposure to a pathogen—using live microorganisms at sublethal doses or inactivated microbes through chemical or physical treatments (e.g., heat shock or formalin)—enhances immune responses and increases resistance to subsequent infections (Melillo et al., 2018; Lanz-Mendoza and Contreras-Garduño, 2022; Montagnani et al., 2024).

Despite invertebrates comprising more than 95% of animal diversity, immune priming has been demonstrated in only a limited number of taxa, including cnidarians, mollusks, and several ecdysozoan orders (Pradeu and Du Pasquier, 2018; Melillo et al., 2018; Low and Chong, 2020; Lanz-Mendoza and Contreras-Garduño, 2022; Montagnani et al., 2024). Among invertebrates, marine bivalves constitute ideal models to investigate immune priming due to their ecological importance and economic relevance (Montagnani et al., 2024). With more than 25,000 described species distributed across marine and estuarine environments (World Register of Marine Species, 2026), bivalves act as ecosystem engineers. Through their filter-feeding activity, they interact intensively with their surrounding environment, filtering large volumes of water—including microorganisms—while removing suspended particles and pollutants, improving water clarity, and contributing to nutrient cycling, sediment stabilization, and blue carbon storage (Vaughn and Hoellein, 2018; Alleway et al., 2025). Beyond their ecological role, bivalves—e.g., clams, oysters, mussels, and scallops—include cornerstone species for global aquaculture and the blue bioeconomy (Naylor et al., 2021; FAO, 2024). However, their intensive filter-feeding behavior also renders them particularly vulnerable to infectious diseases. Vibriosis, caused by pathogenic *Vibrio* species, represents one of the major constraints to sustainable bivalve aquaculture worldwide, leading to recurrent mass mortality events that threaten economic viability. Despite decades of research, effective and environmentally sustainable prophylactic or therapeutic solutions remain lacking (Dubert et al., 2017a; Smolowitz, 2024). Antibiotics are still commonly used in aquaculture settings such as hatcheries to protect early life stages, yet their misuse has accelerated the emergence and dissemination of antimicrobial resistance among *Vibrio* populations, raising serious ecological and public health concerns (Dubert et al., 2016a). Moreover, preventive treatments are difficult to implement in open marine environments, highlighting the urgent need for alternative disease management strategies.

Among pathogenic vibrios, *V. europaeus* has emerged over the last decade as a particularly serious threat to shellfish aquaculture, being responsible for recurrent and massive mortality events in hatcheries in France, Spain, Chile, and the United States—countries that rank among the world's top bivalve producers (Dubert et al., 2017a; Rojas et al., 2021). This bacterial pathogen displays a broad host range, infecting key aquaculture species such as the Manila clam (*Ruditapes philippinarum*), Pacific oyster (*Magallana gigas*), and Chilean scallop (*Argopecten purpuratus*), as well as gastropod mollusks including abalones (*Haliotis* spp.) (Saulnier et al., 2010). Importantly, *V. europaeus* exhibits virulence across multiple developmental stages, from larvae to juveniles (Saulnier et al., 2010; Mersni-Achour et al., 2014; Travers et al., 2014; Prado et al., 2005, 2015; Mersni-Achour et al., 2015; Dubert et al., 2016b, 2017b), making it a highly relevant bacterial model to evaluate immune priming in bivalves.

Despite the ecological and economic importance of marine bivalves, evidence of immune priming in this group remains scarce and taxonomically uneven. To date, immune priming has been demonstrated in a limited number of species, including the Japanese scallop (*Chlamys farreri*) (Cong et al., 2008, 2009; Yue et al., 2013), mussels (*Mytilus galloprovincialis* and *Perna viridis*) (Aleng et al., 2015; Rey-Campos et al., 2019), and predominantly the Pacific oyster, where priming has been extensively studied against bacterial and viral pathogens (Green and Montagnani, 2013; Green et al., 2014, 2016; Zhang et al., 2014; Gerdol and Venier, 2015; Liu et al., 2016; Pauletto et al., 2017; Li et al., 2017; Lafont et al., 2017, 2019, 2020; Liu et al., 2023; Lian et al., 2024). In contrast, clams have been largely overlooked, with only one recent study reporting immune priming in the Manila clam and focusing exclusively on host immune responses to *V. anguillarum* infection (Li et al., 2025).

Critically, most studies in marine invertebrates adopt a host-centric perspective, focusing primarily on host immune responses while overlooking that immunity operates within a complex microbial environment. In insects, host–microbiota interactions play a central role in immune priming, as demonstrated by the loss of priming capacity following microbiota depletion in *Anopheles gambiae* and by the contribution of symbiotic bacteria to shape host immunity and contribute directly to immune priming by inducing immune effectors (e.g., AMPs) or modulating immune signaling pathways (e.g., the Toll pathway) in several taxa (e.g., beetles, moths, honeybees, and cockroaches) (Hernández-Martínez et al., 2010; Futo et al., 2016; Horak et al., 2020; Korša et al., 2022; Lang et al., 2022; Jang et al., 2024; Turner et al., 2024; Keshavarz et al., 2025). In marine invertebrates, however, such tripartite interactions among host, microbiota, and pathogen remain poorly explored and have been investigated exclusively in the Pacific oyster (Fallet et al., 2022). Notably, pathogen dynamics under immune priming pressure have never been examined in marine invertebrates, leaving fundamental questions unresolved regarding how priming affects pathogen persistence and proliferation.

Here, we present the first study about the impact of oral immune priming on the microbiota of the Manila clam, while simultaneously examining the dynamics of the pathogen *V. europaeus*. Using an ecologically realistic experimental framework, we integrate host survival analyses, quantitative pathogen monitoring by quantitative PCR (qPCR), and full-length 16S rRNA gene metabarcoding to characterize microbiota shifts under priming pressure. This integrative approach provides new insights into the role of the microbiota and pathogen behavior in immune priming, advancing our understanding of immune adaptation in marine invertebrates.

## 2 Materials and methods

### 2.1 Model organisms: Manila clam (*Ruditapes philippinarum*) and *Vibrio europaeus*

Experimental challenges were conducted using Manila clam juveniles (*R. philippinarum*, 13 ± 1 mm shell length, 8 months old). A total of 1,000 clams were supplied by Proameixa (Spain) and acclimated prior to the experiment in aquaria at the University of Santiago de Compostela (USC). Clams were maintained until use at room temperature (RT; approximately 19 ± 1 °C) under aeration and regularly fed with EasyBooster25 (Easyreefs, Spain) following the manufacturer's recommendations.

*Vibrio europaeus* CECT 8136^T^ was used to evaluate immune priming in Manila clam. This bacterium was routinely cultured on Tryptone–Soy agar or broth (TSA-2/TSB-2, Condalab, Spain) supplemented with 2% (w/v) NaCl at 25 °C for 24 h. For the challenges described below, bacterial suspensions were prepared in sterile seawater (SSW) from overnight cultures and adjusted to an optical density of 1.0 at 600 nm (OD_600_), corresponding to approximately 10^8^ colony-forming units (CFU) mL^−1^. Serial ten-fold dilutions were performed (i) to confirm bacterial counts by spreading on TSA-2 plates and the concentration was expressed as CFU mL^−1^, and (ii) to adjust the bacterial inoculum to the desired final concentration according to the objectives of each challenge assay.

### 2.2 Immune priming challenge

**Figure 1:**
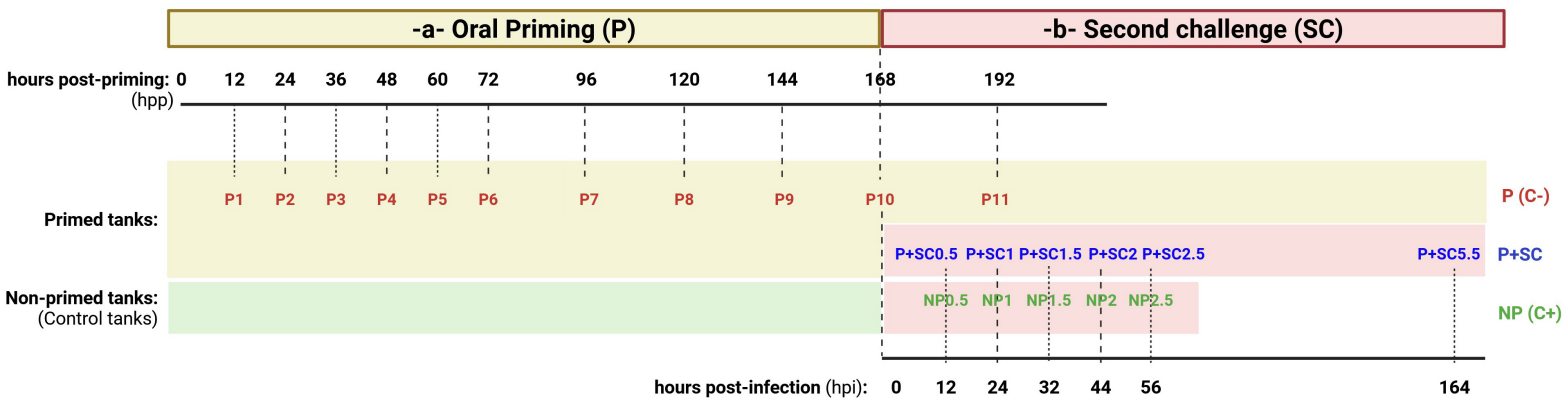
Overview of the experimental design to induce immune priming in the Manila clam. Sampling points are indicated as hours post-priming (hpp) or hours post-infection (hpi). Experimental conditions are represented by colors: **(a)** red, animals primed during the priming stage (P0–P11); samples P10 (0 hpi) and P11 (24 hpi) were included for comparative analyses with those challenged during the second exposure and served as negative controls (P C–); **(b)** second challenge, animals exposed to the bacterial pathogen during the second infection stage—blue, primed animals (P + SC; P + SC0.5 – P + SC5.5); and green, non-primed animals (NP C+; NP0.5–NP2.5).

The experimental design consisted of two consecutive exposures of Manila clam juveniles to the pathogen (*V. europaeus*; Figure 1). Six tanks (tank size: width, 8.5 cm; height, 6.5 cm; length, 12.5 cm; surface area, 106.25 cm^2^; volume, 690.6 cm^3^) were used per condition (three for DNA sampling and three for monitoring survival rates) as follows: (i) Non-primed clams (*n* = 6 tanks) that were infected with the pathogen during the second challenge, used as positive controls (NP C+); (ii) primed clams infected during the second challenge (P + SC; *n* = 6 tanks) to evaluate increased survival associated with immune priming; (iii) primed tanks not infected during the second challenge (*n* = 6 tanks), used as negative controls (P C–) to discard potential negative effects of priming on host survival throughout the experimental period. Briefly, the experimental design included two main steps (Figure 1):

- Oral priming: NP C+ tanks (15 juveniles per tank) contained 100 mL of filtered seawater (FSW; 0.22-μm Nalgene Rapid-Flow, Thermo Fisher, United States). On the other hand, Manila clam juveniles from P C– and P + SC tanks (15 juveniles per tank) were orally primed by immersion in FSW containing *V. europaeus* CECT 8136^T^ at a non-lethal dose (final concentration ~10^6^ CFU mL^−1^). All tanks were kept under aeration for 7 days (168 hours post-priming, hpp), with food supply and water renewal at day 5 (120 hpp).
- Second challenge: after 1 week, clams from both NP (C+) and P + SC tanks were exposed to a lethal dose of *V. europaeus* CECT 8136^T^ (final concentration ~10^7^ CFU mL^−1^) to evaluate the immune priming response, following the infection protocol described by Martinez et al. (2022). P (C–) tanks were not infected and served as negative controls as described above. For infection, juveniles were transferred to infection tanks and immersed in the bacterial suspension for 24 h at room temperature (RT) without aeration (seawater was previously oxygenated to ensure adequate oxygen availability throughout the immersion period) to promote active filtration of the bacteria. After infection, challenged juveniles were removed from the infection tanks and held dry for 8 h at RT to ensure bacterial internalization within the pallial cavity. Finally, the juveniles were transferred to clean tanks containing 200 mL of FSW with aeration. Seawater was renewed every 24 h, and clams were maintained without food throughout the second challenge period.

### 2.3 Survival rates

Survival rates were calculated and expressed as the percentage of surviving individuals (%) during both priming and second challenge step (Figure 1). During the priming phase, survival was recorded at 0, 12, 24, 36, 48, 60, 72, 96, 120, 144, 168, and 192 hours post-priming (hpp) in both primed and non-primed groups (Figure 1). After the second challenge, mortality was recorded at 24, 32, 44, 56, 68, and 80 hpi. Because high survival was observed in primed clams following the second challenge, survival rate was also evaluated at 164 hpi (Figure 1). Clams were considered dead when their valves remained open, or moribund when siphons failed to retract upon stimulation. Dead and moribund juveniles were immediately removed from the tanks. The bacterial pathogen (*V. europaeus*) was routinely re-isolated on Thiosulfate-citrate-bile salts-sucrose agar (TCBS, Oxoid, United Kingdom) from moribund or dead juveniles in order to fulfill Koch's postulates and confirm the causative role of the pathogen.

Survival rates were plotted using Kaplan–Meier survival curves generated in GraphPad software (United States). Statistical comparisons among curves were performed using log-rank (Mantel–Cox) tests for trend in multiple comparisons analysis, with the significance level set at *p* < 0.05.

### 2.4 Sample collection for DNA extraction

Samples for total DNA extraction were collected at the time points indicated above (Figure 1). During the priming phase, eleven samples (P1–P11) were collected from 0 to 192 hpp (Figure 1). Sample P11 (equivalent to 24 hpi), obtained from primed clams that were not infected during the second challenge, was used as a negative control of priming for comparison with infected samples (Figure 1). After the second challenge, five samples were collected at 12, 24, 32, 44, and 56 hpi from primed clams, including both previously primed (P + SC0.5 – P + SC2.5) and non-primed individuals (C+; NP0.5–NP2.5). Additionally, a final sample for primed clams was collected at 164 hpi (P + SC5.5).

One juvenile was randomly collected from each of the three tanks used for DNA sampling to minimize potential tank-related biases. Thus, a total of three juveniles were collected per sampling point and processed together as a pooled sample. Clam shells were individually removed using sterile scalpel blades, and the whole bodies were lyophilized and subsequently homogenized by bead beating using a Mixer Mill MM400 (Retsch, Germany). The resulting clam powder was stored at −80 °C until further use. Total DNA was extracted from approximately 1.5 mg of each pooled sample using the DNeasy Blood and Tissue Kit (QIAGEN, Netherlands) according to the manufacturer's instructions. The concentration and purity of the extracted DNA were assessed using a NanoDrop One spectrophotometer (Thermo Scientific, United States), a Qubit fluorometer (Thermo Scientific, United States), and capillary electrophoresis using an Agilent BioAnalyzer 2100 (Agilent Technologies, United States).

### 2.5 Quantification of *Vibrio europaeus* for pathogen dynamics

Total DNA was used to evaluate pathogen dynamics during both the priming and second challenge exposures (Figure 1). The concentration of *V. europaeus* was quantified using a *ktrA*-based real-time quantitative PCR (qPCR) TaqMan assay described by Rey-Varela et al. (2026), providing a highly-specific protocol for the detection of *V. europaeus*. Primers, TaqMan probe, and standard DNA nucleotide sequences are provided in Supplementary Table S1. Each sample was analyzed in quadruplicate (two undiluted and two ten-fold diluted).

Briefly, qPCR reactions were carried out in 20 μL containing 2 μL of template DNA, 10 μL Luna Universal Probe qPCR Master Mix (New England Biolabs, United States), 0.4 μL Antarctic Thermolabile UDG (New England Biolabs, United States), 0.4 μL probe (10 μM stock; final concentration 0.2 μM), 0.8 μL of each primer (10 μM stock; final concentration 0.4 μM), and 6.4 μL of molecular-grade water. Reactions were run in 96-well plates sealed with heat-bonding film on a C1000 Touch thermocycler coupled to a CFX96 Touch Real-Time PCR Detection System (Bio-Rad, United States). Thermal cycling conditions consisted of an initial step at 25 °C for 10 min, followed by one cycle at 95 °C for 1 min, and 40 amplification cycles at 95 °C for 15 s, 55 °C for 15 s, and 68 °C for 10 s.

A standard curve was generated to determine the assay sensitivity, limit of detection and quantification, and amplification efficiency, using serial ten-fold dilutions of standard DNA (Supplementary Table S1). qPCR results were expressed as *V. europaeus* genome copies per milligram of whole-body clam tissue (copies mg^−1^).

### 2.6 Library preparation and full-length 16S rRNA amplicon sequencing

PCR amplification, library preparation, and sequencing were performed by Novogene (United Kingdom). For each sample, three independent amplification rounds were carried out using total DNA to amplify the full-length 16S rRNA gene with barcoded forward (27F: 5′-AGAGTTTGATCCTGGCTCAG-3′) and reverse primers (1510R: 5′-GGTTACCTTGTTACGACTT-3′), following the protocol described in “Preparing Kinnex™ libraries from 16S rRNA amplicons” (Pacific Biosciences, United States). The Kinnex 16S rRNA kit was used to increase throughput on PacBio long-read sequencers by applying a concatenation method that joins genomic DNA molecules (MAS-Seq) into longer fragments (Al'Khafaji et al., 2024).

Thermal cycling conditions consisted of an initial denaturation at 95 °C for 3 min, followed by 20 amplification cycles at 98 °C for 20 s, 57 °C for 30 s, and 72 °C for 75 s, with a final extension at 72 °C for 5 min, using 2 × KAPA HiFi HotStart Ready Mix polymerase (Roche, Switzerland). The expected amplicon size (~1,500 bp) was verified by electrophoresis on a 1% (w/v) agarose gel (Lonza, Switzerland) stained with Gel Red Nucleic Acid Gel Stain (Biotium, United States). Three replicates were pooled equimolarly and cleaned using SMRTbell Cleanup beads (Pacific Biosciences, United States) according to the manufacturer's protocol. Cleaned PCR products were quantified using a Qubit fluorometer (Invitrogen, United States) with the 1× dsDNA HS Assay kit (Invitrogen, United States). Subsequently, 16S amplicons were processed to prepare the Kinnex™ libraries following the PacBio protocol. This step adds Kinnex adapters to the ends of barcoded full-length 16S amplicons, enabling concatenation of PCR products to approximately 19 kb. Library concentration was verified with a Qubit fluorometer, and quantified libraries were pooled and sequenced on a PacBio Sequel II/IIe system (Pacific Biosciences, United States), according to effective library concentration and data amount required.

### 2.7 Microbiome taxonomical annotation

The PacBio BAM file was split according to barcodes and filtered to obtain clean data. Briefly, PacBio offline data were exported to BAM format files. The Lima software was used to distinguish the data of each sample based on barcode sequences and to save all sample sequences in BAM format. Then, CCS (SMRT Link v7.0) was used to correct the sequences, with a correction parameter of CCS = 3 and a minimum accuracy of 0.99. Sequences with lengths shorter than 1340 and longer than 1640 were removed and stored in FASTQ and FASTA formats. Subsequently, SSR filtration was performed, and the primers were removed using cutadapt to filter out sequences containing >8 consecutive identical bases. The reads obtained after the above processing were considered the final valid data (Clean Reads) and are shown in Supplementary Table S2.

The obtained demultiplexed PacBio FASTQ sequence files were imported into QIIME2 (v2020.6). Denoising was performed with the DADA2 module (default parameters) in the QIIME2 software (v2020.6) to obtain initial amplicon sequence variants (ASVs; default: DADA2), and then ASVs with an abundance lower than five were filtered out (Li et al., 2020). The ASVs were compared with the SILVA 138 database (Quast et al., 2012). Rarefied ASV abundance was used for all downstream analyses (rarefied to 8,441 reads). ASVs with fewer than 20 counts across all samples or present in only one condition were filtered out. The 150 most abundant ASVs were taxonomically assigned using EZBioCloud 16S-based ID (Chalita et al., 2024). ASVs assigned to the same taxon were subsequently collapsed into a single representative ASV, as detailed in Supplementary Table S3.

The Shannon index for alpha diversity (within samples) was calculated for all samples with the R library vegan (v2.6-10) using the diversity function with index = “shannon” and default values. Bray–Curtis dissimilarity and Jaccard distance was calculated to obtain beta diversity (between samples) with the R library vegan (v2.6-10) using the vegdist function with distance = “bray” or distance = “jaccard,” respectively, and default values. Nonmetric multidimensional scaling (NMDS) was calculated from Bray–Curtis dissimilarity and Jaccard distance using the R library vegan (v2.6-10) with the metaMDS function, indicating distance = “bray” and *k* = 2.

### 2.8 Normalization and clustering

Pseudocounts were obtained by adding one to all the rarefied ASV counts. Rarefied ASV pseudocounts were normalized using the centered log-ratio (CLR) transformation with the R library compositions (v2.0-8) using the clr command with default values. To calculate the clusters, the distance between CLR-normalized values was calculated using the R library compositions (v2.0-8) with the dist command and method = “euclidean,” using default values. The clustering was calculated using the hclust command with method = “ward.D2,” using default values. The hierarchical clustering was divided using the cutree command with *k* = 6. This process was performed following two approaches by comparisons between: (i) the priming phase (P9–P11), the second challenge (SL2.5–SL5.5) and (NP C+: NP0.5–NP2.5); and (ii) all the time points from the priming phase (P1–P11).

## 3 Results

### 3.1 Oral priming effectively protects the Manila clam against the bacterial pathogen *V. europaeus*

No mortalities were observed during the priming phase and survival trends were consistent across all six tanks (three used for DNA sampling and three for monitoring survival rates), indicating that no tank effect was detected along the entire immune priming challenge.

**Figure 2:**
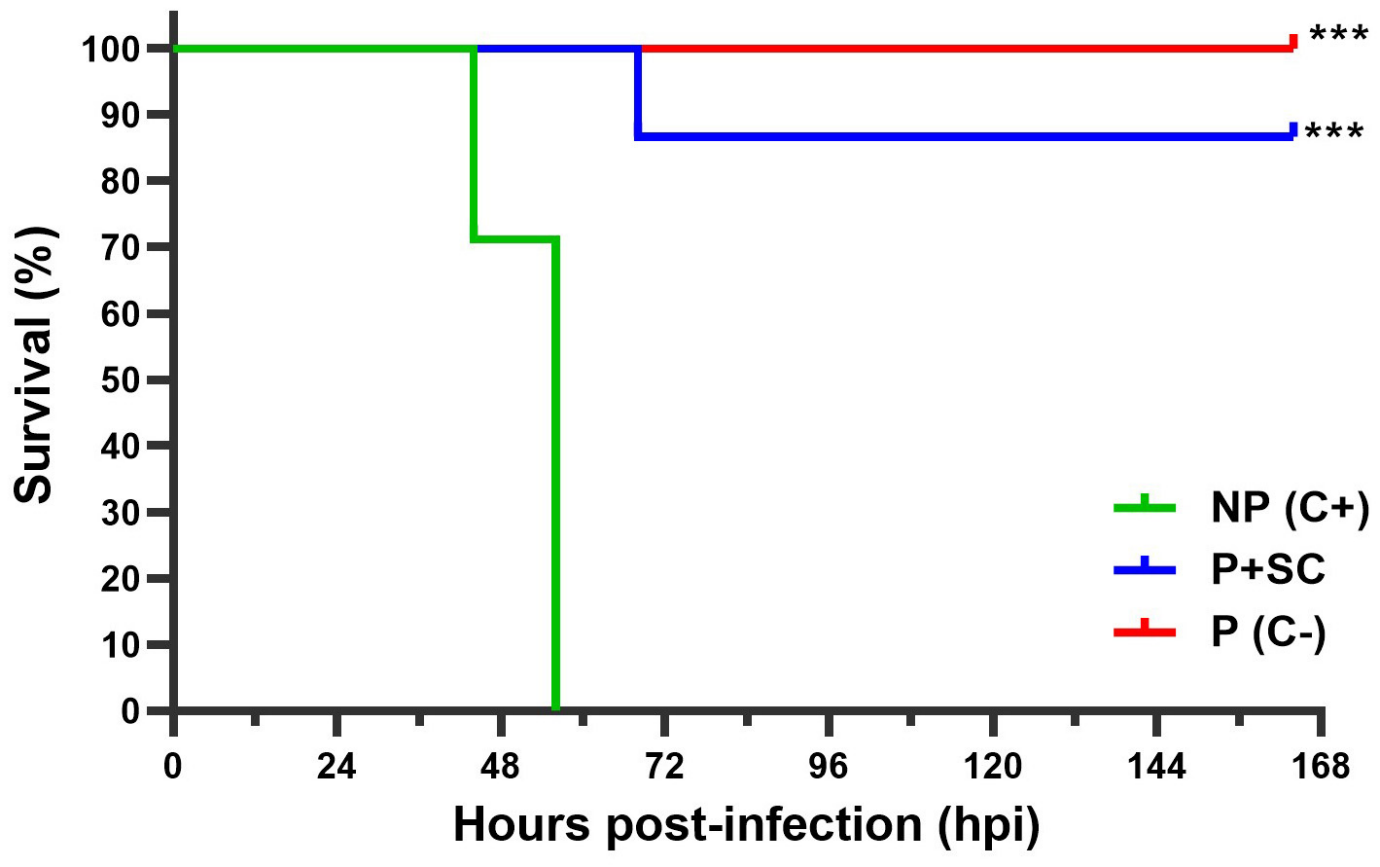
Kaplan–Meier survival curves (%) of Manila clam juveniles following the second challenge. Primed clams (P + SC, blue curve) and non-primed clams (NP C+, green curve) were infected with *V. europaeus* at a lethal dose (10^7^ CFU mL^−1^). Primed clams that were non-challenged with the pathogen served as negative controls (P C–, red curve). Asterisks indicate significant differences with the non-primed group (NP C+; *p* < 0.001).

However, a significant difference in survival (*p* < 0.0001) was detected between primed (P + SC) and non-primed (NP C+) clams following the second challenge with *V. europaeus* at lethal dose (Figure 2). NP clams (C+) exhibited 100% mortality within 56 hours post-infection (hpi), whereas P + SC clams showed only minor mortalities starting at 68 hpi and maintained a stable survival rate of approximately 87% thereafter (Figure 2). Among primed clams, significant differences (*p* = 0.0117) were observed between those infected in the second challenge (P + SC) and non-primed (NP C–), which maintained 100% survival throughout the experiment (Figure 2). Overall, these results provide the first phenotypic evidence of oral immune priming in Manila clams, demonstrating effective protection against bacterial infection by *V. europaeus*.

### 3.2 Immune priming modulates pathogen dynamics, reducing pathogen concentrations to similar levels after priming and the second challenge

Prior to the quantification of *V. europaeus*, the standard curve *y* = −*3.324* × *log(X)* + *40.02* was generated. The assay demonstrated high sensitivity, with a limit of detection (LOD) of 3.93 copies per reaction and a limit of quantification (LOQ) of 19.6 copies per reaction. Amplification efficiency, calculated from the slope (*R*^2^ = 0.989), was 99.9%, confirming the robustness and reliability of the qPCR protocol used.

**Figure 3:**
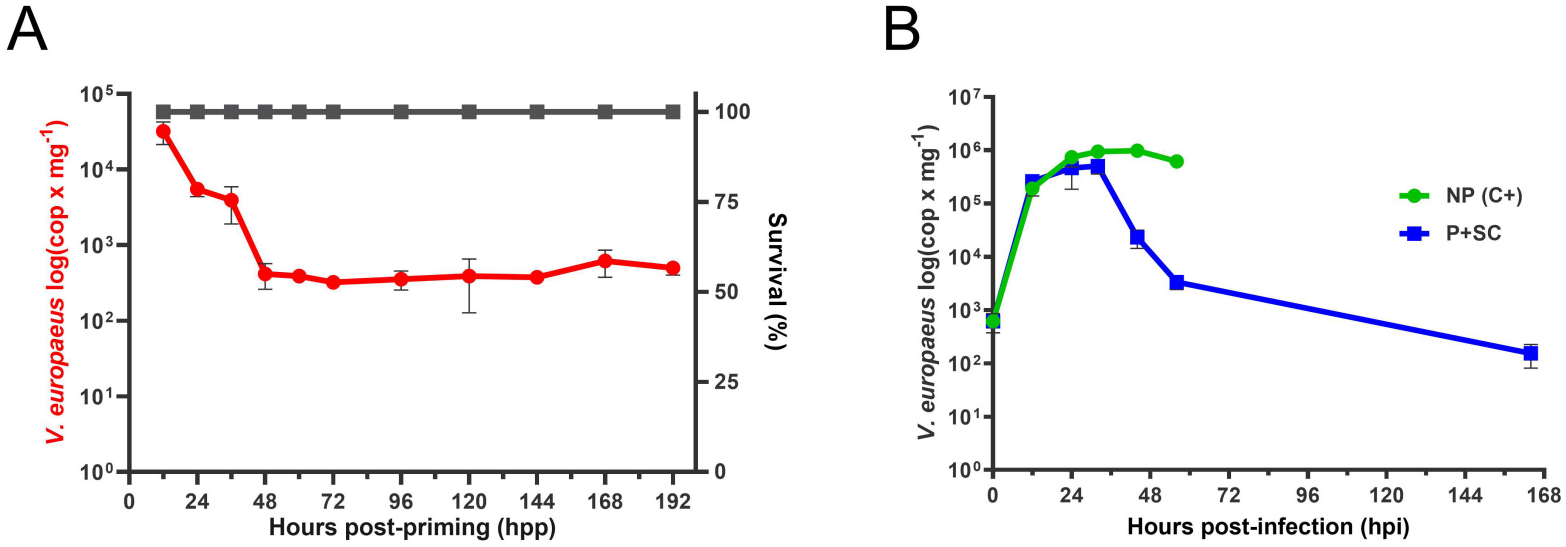
Pathogen dynamics during the priming phase **(A)** and second challenge **(B)**. **(A)** Quantification of *Vibrio europaeus* expressed as genome copies per milligram of pooled clam tissue determined by qPCR (Rey-Varela et al., 2026). The red line represents pathogen concentrations during oral priming. The gray line represents survival during the priming phase, in which no mortalities were detected. **(B)** Pathogen concentration following the second challenge in pooled primed clams (P + SC, blue line) and pooled non-primed clams (NP C+, green line). Each sample was analyzed in quadruplicate (two undiluted and two 10-fold diluted reactions). Bars represent the standard deviation among technical replicates.

During the priming phase (Figure 3A), the concentration of *V. europaeus* decreased by two orders of magnitude—from 3.21 × 10^4^ to 4.15 × 10^2^ copies mg^−1^–within the first 48 hours post-priming (hpp). Pathogen concentrations remained stable thereafter throughout the experiment and no mortalities were detected at any time.

After the second challenge, pathogen concentrations increased in primed clams (P + SC) during the first 32 hpi, reaching a peak of 5.07 × 10^5^ copies mg^−1^ (Figure 3B). From this time point onward, P + SC clams bacterial concentrations decreased by approximately two orders of magnitude, reaching 3.37 × 10^3^ copies mg^−1^ at 56 hpi. A further decrease was observed at 164 hpi, with pathogen levels reaching 1.54 × 10^2^ copies mg^−1^, comparable to those measured during the priming phase. In contrast, non-primed clams (NP C+) were unable to reduce bacterial concentrations below 10^5^ copies mg^−1^ after 32 hpi. This pattern coincided with the onset of high mortality observed in this group and preceded the complete mortality observed (Figures 2, 3B). It is important to remark that concentrations in negative controls P10 (168 hpp or 0 hpi) and P11 (192 hpp or 24 hpi) were stable around 10^2^ copies mg^−1^ as observed in the priming phase (Figure 3A).

These results suggest that: (i) the capacity of primed clams to control and reduce *V. europaeus* concentrations below the mortality threshold (~10^5^ copies mg^−1^ after 32 hpi) inferred from non-primed animals after second challenge, and (ii) the persistence of the pathogen at low concentrations in primed clams without apparent harm to the host.

### 3.3 Beta diversity demonstrates the establishment of a specific community composition associated with immune priming

Alpha diversity metrics suggested temporal fluctuations across the experiment (Supplementary Figure S1), with an apparent decrease during the early priming phase, followed by increased variability during the second challenge and a subsequent recovery toward diversity levels comparable to those observed at the beginning of the experiment. However, additional samples should be analyzed to confirm this hypothesis and to overcome limitations associated with the experimental design.

**Figure 4:**
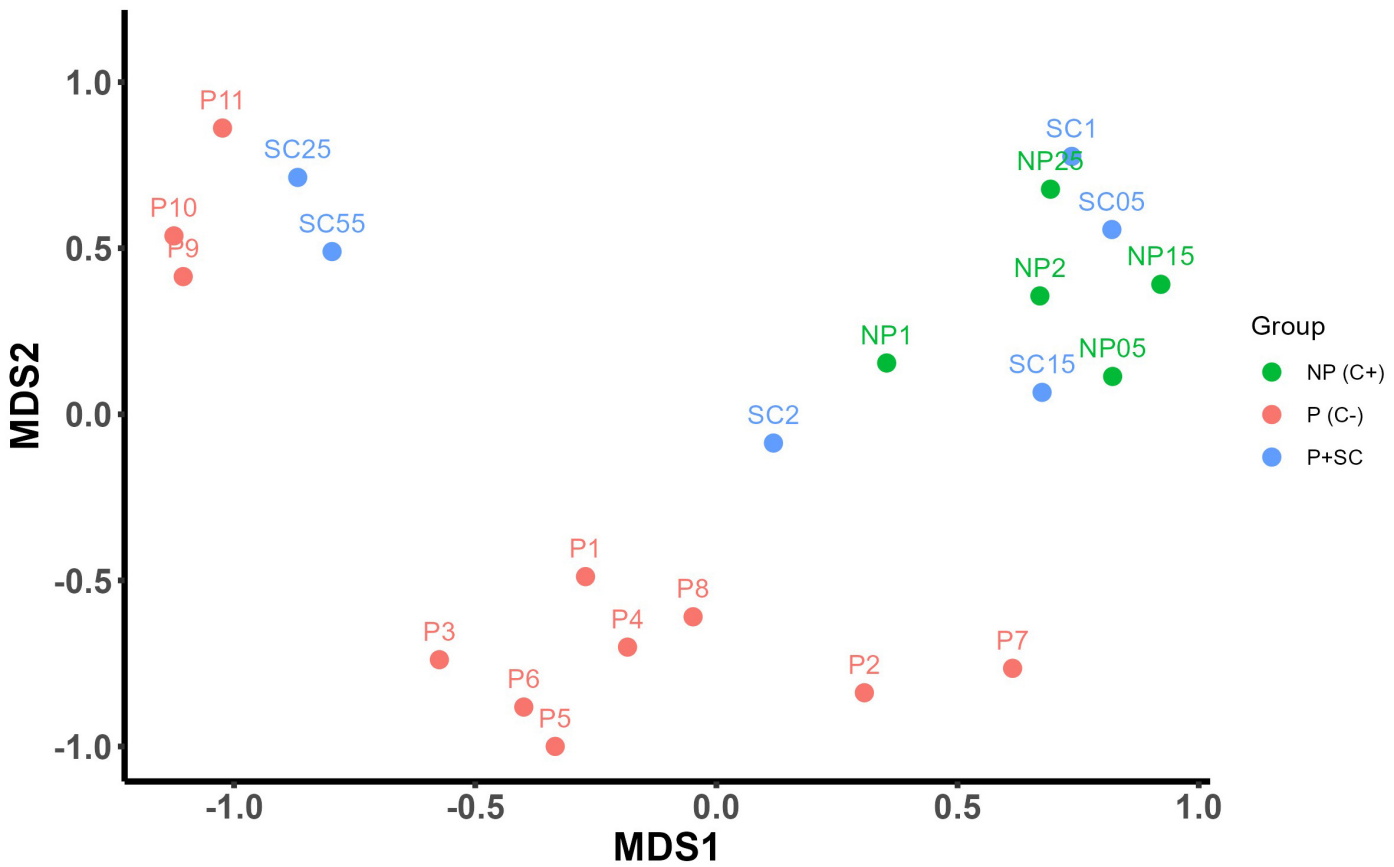
Beta diversity analyses of the Manila clam microbiota. Beta diversity visualized by multidimensional scaling (MDS) based on Bray–Curtis dissimilarities. Pooled samples collected during the priming phase (P1–P11, red) and the second challenge (P + SC, blue and NP, green) are shown.

To assess differences in microbiota composition among samples, Bray–Curtis dissimilarities and Jaccard distance were calculated and visualized using multidimensional scaling (MDS; Figure 4 and Supplementary Figure S2, respectively). During the priming phase, samples collected between 12 and 120 hpp (P1–P8) clustered closely together. From 144 hpp onward (P9), microbiota composition shifted and subsequently stabilized, with samples P9, P10, and P11 clustering together (Figure 4 and Supplementary Figure S2).

Beta diversity analyses further revealed distinct compositional patterns between the priming phase and the second challenge. During the first 32 hours post-infection (hpi), primed (P + SC: SC0.5–SC1.5) and non-primed clams (NP C+: NP0.5–NP1.5) clustered together, coinciding with a sharp increase in pathogen abundance (~10^5^ copies mg^−1^; Figure 3B). Non-primed samples collected at 44 and 56 hpi (NP C+: NP2 and NP2.5) also grouped within this cluster (Figure 4), consistent with peak mortality events (Figure 2). However, from 44 hpi onward, only primed clams (P + SC: SC2–SC5.5) progressively shifted away from this cluster and grouped with samples collected at the end of the priming phase, clustering again with samples P9–P11, coinciding with a two-log reduction in pathogen load (Figures 3B, 4 and Supplementary Figure S2).

### 3.4 Immune priming promotes a specific and stable microbiota

**Figure 5:**
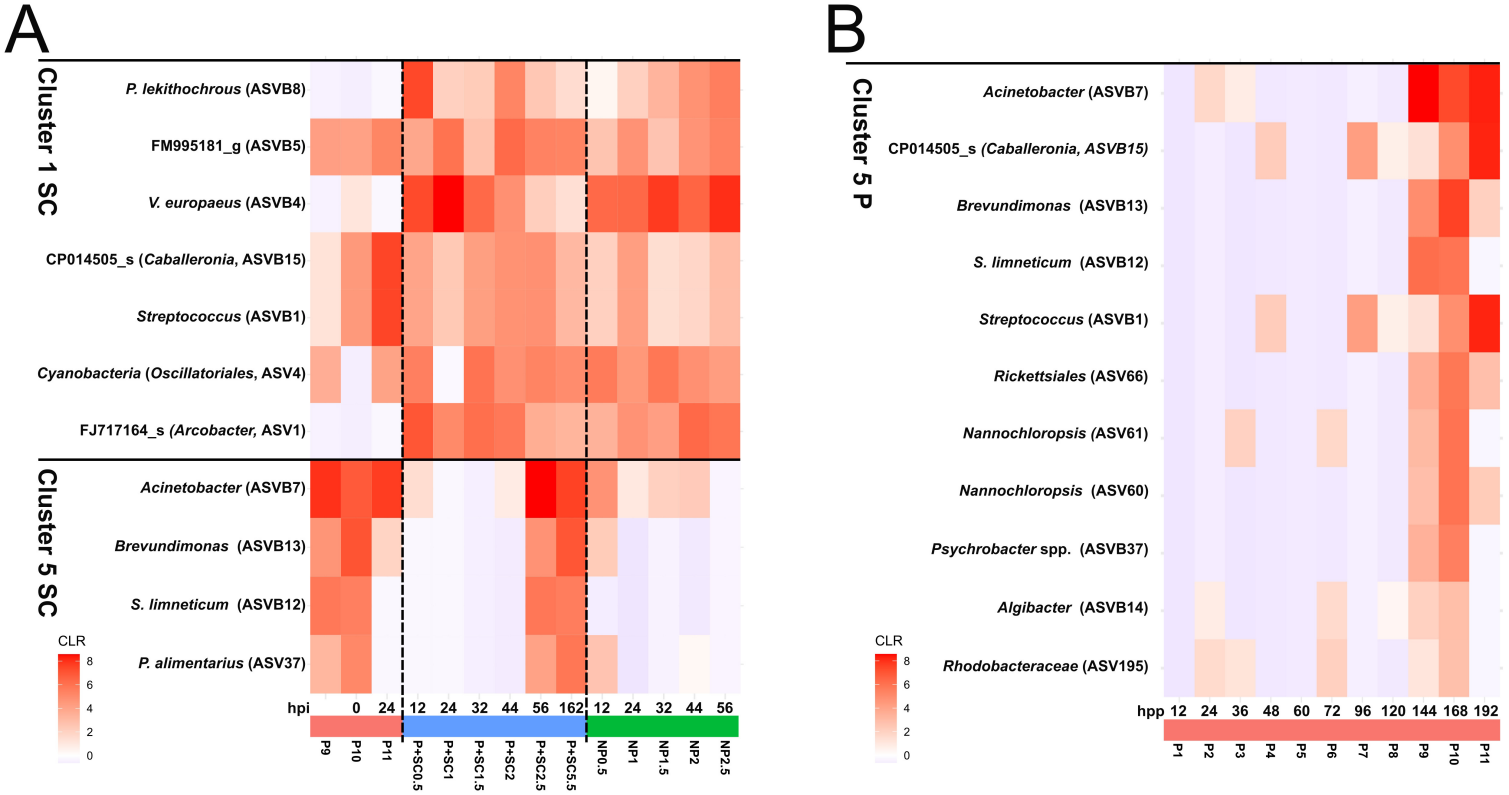
Clustering analysis of bacterial taxa associated to immune priming in Manila clam during the second challenge **(A)** and priming phase **(B)**. Rarefied amplicon sequence variant (ASV) counts were normalized using the centered log-ratio (CLR) transformation. Clustering analysis based on CLR-normalized ASV abundances identified six major clusters of bacterial taxa across the analyzed samples. **(A)** Heatmap showing the bacterial composition of Cluster 1SC and Cluster 5SC across the experimental groups (P9–P11, P + SC and NP), which accounted for most of the variation observed in the beta diversity analyses. Clusters 2SC, 3SC, 4SC and 6SC are shown in the Supplementary material. **(B)** Heatmap showing the bacterial composition of Cluster 5P across the priming phase (P1–P11).

Clustering analysis was performed to identify bacterial taxa specifically associated with immune priming. Two complementary approaches were applied: (i) taxa associated with samples that grouped together in the beta diversity plot at the end of both the priming phase (P9–P11) and the second challenge (SL2.5–SL5.5), whose microbiota was compared with non-primed clams (NP C+: NP0.5–NP2.5; Figures 4, 5A); and (ii) bacterial composition across the priming phase (P1–P11; Figures 4, 5B). After centered log-ratio (CLR) normalization and hierarchical clustering, the elbow method indicated six clusters as the optimal number of clusters for the first approach (those cluster are indicated by the suffix SC) (Supplementary Figure S3).

The first approach (Figure 4) revealed that Clusters 1SC and 5SC accounted for most of the variation observed in beta diversity analyses. In Cluster 5SC, primed clams at the end of the second challenge (P + SC2.5 and P + SC5.5) exhibited a characteristic microbiota dominated by Alphaproteobacteria, including *Sphingobium limneticum* (ASVB12) and *Brevundimonas* species (*B. huaxiensis/B. vesicularis/B. nasdae/B. fontaquae/B. intermedia*; ASVB13), as well as Gammaproteobacteria belonging to the family *Moraxellaceae*, such as *Acinetobacter* (*A. lwoffii/Prolinoborus fasciculus/A. pecorum*; ASVB7) and *Psychrobacter alimentarius* (ASV37). These taxa were also detected in primed clams at the end of the priming phase (P9–P10) and at the onset of the second challenge (P10, 0 hpi; Figure 5A). *Acinetobacter* and *Brevundimonas* were additionally detected in the priming negative control at 12 hpi (P11; Figure 5A). Members of Cluster 5SC were absent or progressively declined in NP clams, supporting the hypothesis that this cluster is clearly associated with the immune priming response.

In Cluster 1SC, several taxa were not detected in primed clams during the priming phase but appeared during the second challenge, likely in association with infection (Figure 5A). Excluding the pathogen *V. europaeus* (ASVB4), these taxa increased in non-primed clams (NP C+: NP0.5–NP2.5) and decreased in primed clams (P + SC: P + SC0.5 – P + SC5.5), and included members of the family *Arcobacteraceae*, such as *Poseidonibacter lekithochrous* (ASVB8) and an unidentified *Arcobacter* species (FJ717164_s; ASV1; Figure 5A). Other taxa assigned to Cluster 1SC were detected in primed (P) clams during the priming phase as well as in primed (P + SC) and non-primed (NP C+) clams during the second challenge (Figure 5A). These included *Streptococcus* (*S. thermophilus/S. salivarius/S. vestibularis*; ASVB1), an unidentified bacterium such as FM995181_g (ASVB5), CP014505_s belonging to the genus *Caballeronia* (ASVB15), and an unknown cyanobacterium assigned to the order *Oscillatoriales* (ASV4; Figure 5A). Similar patterns were observed for several taxa in Cluster 4SC, including *Cutibacterium acnes* (ASVB9), an unidentified *Oceaniferula species* (GQ262724_s; ASV32), *Cellulophaga* (ASV50), and *Phaeobacter* (ASV48; Supplementary Figure S4). Clusters 3SC and 6SC (Supplementary Figures S5, S6) contained taxa associated with the elevated alpha diversity observed in NP clams at 12 hpi (Cluster 3) and 24 hpi (Cluster 6), suggesting that both clusters are clearly associated with infection and discarding their association with the immune priming state. Cluster 2SC comprised taxa sporadically detected across samples (Supplementary Figure S7).

The second approach (clusters indicated by the suffix P) confirmed the taxa associated with immune priming. Cluster 5P included several species that increased throughout the priming phase and were particularly abundant in the final samples (P9–P11; Figure 5B). These included four species previously identified in Cluster 5SC of the first clustering approach such as *Acinetobacter* (*A. lwoffii/Prolinoborus fasciculus/A. pecorum*; ASVB7), *Brevundimonas* species (*B. huaxiensis/B. vesicularis/B. nasdae/B. fontaquae/B. intermedia*; ASVB13), *Sphingobium limneticum* (ASVB12) and *Psychrobacter alimentarius* (ASV37).

Cluster 5P included also the species *Streptococcus* (*S. thermophilus/S. salivarius/S. vestibularis*; ASVB1) and *Caballeronia* (ASVB15; Figure 5B), previously found in the Cluster 1SC. However, these two taxa were detected in all samples from 144 hpp to 162 hpi (Figure 5), including both primed and non-primed juveniles, suggesting that their proliferation was associated with the prolonged duration of the experiment and was therefore likely promoted by the experimental conditions rather than by immune priming.

Overall, both clustering analyses revealed the proliferation of a specific and stable microbiota associated with immune priming, consistently detected in primed clams during both the priming phase and the second challenge, as also supported by the beta diversity analyses (Figures 4, 5).

## 4 Discussion

This study provides the first phenotypic evidence of successful oral immune priming in the Manila clam (*R. philippinarum*), demonstrating a robust protection against the bacterial pathogen *V. europaeus*. These findings confirm the existence and persistence of innate immune memory in a key aquaculture bivalve such as the Manila clam, extending previous evidence reported by others (Li et al., 2025).

Immune priming in bivalves has been mostly induced by injection, including the only study reported in Manila clams to date (Wang et al., 2013; Zhang et al., 2014; Rey-Campos et al., 2019; Lafont et al., 2017, 2020; Li et al., 2025). In contrast, the present study demonstrates that the oral route can effectively induce immune priming. This is particularly relevant because bivalves are filter feeders, and oral exposure represents their natural route of infection in the environment (Dubert et al., 2017a). Moreover, injection-based approaches induce physiological and immunological biases, as tissue injury caused by injection can itself trigger immune activation. Indeed, wounding has been shown to induce strong immune responses independently of pathogen exposure (Johnston and Rolff, 2013; Behrens et al., 2014). In the Manila clam, oral priming resulted in higher survival following the secondary challenge (87%) than previously reported using injection-based priming (82%) (Li et al., 2025). Notably, no mortality was observed during the priming phase in the oral model, whereas injection-based priming resulted in substantial mortality 69% survival during this phase, likely due to wounding stress and the injection of high bacterial concentrations (5 × 10^7^ CFU mL^−1^). Together, these observations indicate that the oral route provides a more physiologically relevant and less invasive strategy for inducing immune priming in bivalves, while minimizing host stress.

In addition, many immune priming studies have relied on inactivated pathogens (i.e., dead microorganisms) to induce priming responses (Yue et al., 2013; Wang et al., 2013; Zhang et al., 2014; Liu et al., 2016; Li et al., 2017). However, in natural environments, immune priming is expected to occur in response to live pathogens. The use of live bacteria is therefore essential to evaluate pathogen dynamics under priming pressure. By combining oral exposure with live pathogens, our experimental design provides an ecologically realistic framework to investigate immune priming in marine bivalves and to gain a more accurate understanding of how this phenomenon operates under natural conditions.

From an applied perspective, oral immunization also offers clear practical advantages: it allows the potential immunization of large numbers of animals without the need for individual handling, making it particularly suitable for large-scale aquaculture operations. Despite decades of intensive mollusk production, no effective and eco-friendly prophylactic or therapeutic strategies have yet been established to control bacterial pathogens in shellfish aquaculture (Dubert et al., 2017b; Smolowitz, 2024). Immune priming therefore emerges as a promising alternative to address this gap. The survival rates achieved in this study are comparable to—or higher than—those reported in other immune priming experiments involving different mollusk hosts and *Vibrio* species (Montagnani et al., 2024). Our results provide additional evidence supporting the development of vaccination-like strategies based on immune priming as a sustainable approach to disease management in bivalve aquaculture.

This study demonstrates for the first time in a marine bivalve that immune priming is accompanied by profound shifts in the host-associated microbiota, affecting both bacterial diversity and community composition. Alpha diversity suggests a tendency to decrease during the priming phase, followed by increased variability during the early time points of the second challenge, with values later approaching those observed at the end of the priming phase. Although these observations are descriptive due to the limitations of the experimental design, this pattern is consistent with the possibility that microbiota associated with the primed state may contribute to limiting early community disruption during pathogen proliferation. Beta diversity analyses further supported the establishment of a specific and stable bacterial community associated with the immune-priming state. Following the acute phase of infection, primed clams (P + SC) progressively returned to the microbial structure established at the end of the priming phase (P9–P11), a compositional recovery that coincided with a two-order-of-magnitude reduction in pathogen burden.

By specifically examining microbiota dynamics under priming conditions, we demonstrate that immune priming is associated with the establishment of a specific microbiota composition in the Manila clam. Interactions between microbiota and immune priming have been extensively studied in insects but remain largely overlooked in marine mollusks. In insects, host–microbiota interactions play critical roles in activating immune priming. For instance, elimination of the gut microbiota in *Anopheles gambiae* abolishes immune priming against *Plasmodium falciparum* (Hernández-Martínez et al., 2010). In other insects—including beetles, moths, honeybees, and cockroaches—symbiotic microorganisms can shape host immunity and contribute directly to immune priming by inducing immune effectors (e.g., AMPs) or modulating immune signaling pathways (e.g., the Toll pathway) (Futo et al., 2016; Horak et al., 2020; Korša et al., 2022; Lang et al., 2022; Jang et al., 2024; Turner et al., 2024; Keshavarz et al., 2025). In marine invertebrates, evidence for such interactions is scarce. To date, the only reported example involves the Pacific oyster, in which exposure to non-infectious environmental microbiota during early larval stages induced a systemic immune response that conferred disease protection (Fallet et al., 2022). While previous studies in mollusks have primarily focused on host immune responses (Montagnani et al., 2024), our findings demonstrated the re-emergence of ASVs belonging to Cluster 5SC after the acute stage of the second challenge, supporting a strong association between a specific microbiota composition and immune priming in marine bivalves. Cluster 5SC included taxa such as *Acinetobacter* and *Psychrobacter alimentarius*, both genera with isolates that showed antimicrobial activity against *Vibrio* spp. and against *Aeromonas hydrophila* (Ramírez et al., 2020). These observations highlight the need for future studies aimed at isolating representative bacterial taxa associated with immune priming to experimentally assess their causal role in host protection.

Cluster 1SC included taxa strongly associated with the progression of the infection in non-primed clams. In these animals, the pathogen *V. europaeus* proliferated rapidly throughout the second challenge, together with other co-infecting taxa, particularly *Arcobacter* spp. In contrast, primed clams exhibited the opposite pattern, with reduced proliferation of both *V. europaeus* and *Arcobacter*. Members of the genus *Arcobacter* have been identified, together with *Vibrio*, as pathobionts playing a major role in the development of Pacific oyster mortality syndrome (POMS), a major threat to the aquaculture industry (Clerissi et al., 2023). POMS is characterized by profound alterations of the oyster microbiota following infection with the OsHV-1 μVar virus. Viral infection induces an immunocompromised state that triggers microbiota dysbiosis and subsequent bacteremia, ultimately leading to oyster death. This fatal dysbiosis is accompanied by invasion of connective tissues by opportunistic bacteria—particularly *Vibrio* and *Arcobacter*—which are consistently associated with mortality events (Clerissi et al., 2023). Although members of the genus *Arcobacter* are recognized as human pathogens linked to gastroenteritis associated with the consumption of raw bivalves, their role in bivalve pathogenesis remains poorly understood (Mottola et al., 2016; Ramees et al., 2017). For instance, *Poseidonibacter lekithochrous*, a member of the *Arcobacteraceae* associated here with the disease phenotype in non-primed clams, was previously reported by our group as not being associated with mortality events in shellfish hatcheries (Diéguez et al., 2017). These contrasting observations highlight the need for further studies using culture-dependent approaches and subsequent infection challenges to clarify the roles of *Arcobacteraceae* members during infection.

This study is also pioneering in examining pathogen dynamics under immune priming pressure in a marine bivalve. Notably, *V europaeus* was detected by qPCR at low concentrations in primed clams. Host responses to infection, including resistance and tolerance, are known to play key roles in shaping pathogen virulence evolution. In this context, a recent study provided the first experimental evidence that immune priming can influence pathogen evolution in insects, using the red flour beetle (*Tribolium castaneum*) as a model (Korša et al., 2025). Those authors revealed an increased activity in mobile genetic elements harbored by the pathogen (*Bacillus thuringiensis tenebrionis*), including prophages and plasmids, with variations in a virulence-related plasmid encoding the Cry toxin. This highlights that immune priming can promote diversity in pathogen traits, which may favor adaptation to variable environments. This report underscores the importance of considering pathogen persistence and evolutionary responses when developing immune priming-based strategies. Such considerations are particularly relevant for applied contexts, including medicine, aquaculture, pest control, and insect mass production, where immune priming may impose selective pressures on pathogens with long-term ecological and evolutionary consequences.

A limitation of this study is that sequencing data per sampling point were obtained from a single DNA extraction rather from biological replicates. Specifically, microbiota was obtained from pooled tissues of three individuals, and PCR replicates were subsequently combined prior to sequencing. Consequently, alpha diversity metrics should be interpreted as descriptive temporal trends rather than statistically supported differences, although this pooling strategy integrates both biological and technical variability within each time point.

This study validates an ecologically realistic approach to induce immune protection in the Manila clam and demonstrates that immune priming cannot be fully evaluated through a host-centric perspective alone. By integrating host survival, pathogen dynamics, and microbiota composition, our results reveal that immune priming is accompanied by coordinated changes in both the pathogen and the host-associated microbiota, which together shape the outcome of infection. These findings highlight the necessity of incorporating microbiota and pathogen responses into immune priming research and support a conceptual shift toward a holistic framework based on tripartite interactions among the host immune system, its microbiota, and invading pathogens. Adopting such an integrated perspective will be essential to unravel the mechanisms underlying immune priming and to develop effective, sustainable disease-management strategies in bivalve aquaculture and other applied systems.

## Data Availability

Raw reads were deposited on the NCBI Sequence Read Archive under BioProject PRJNA1358957. The ASV table with counts for each experimental condition is provided in Supplementary material.
