# Supplementary material for: Immune priming reshapes the microbiota and modulates pathogen dynamics in the Manila clam (Ruditapes philippinarum)
Source: Front Microbiol. 2026 Apr 10;17:1803024. doi: 10.3389/fmicb.2026.1803024 (PMC13106551; doi:10.3389/fmicb.2026.1803024)
Supplement: Supplementary file 1 [file Presentation_1.pdf]

Supplementary Material

1 Supplementary Tables

Table S1. Primers, probe and DNA standard used for the *ktr*A-based qPCR assay (Rey-Varela et al., 2026).

**Primers**	**Sequence (5'-3')**	**Amplicon size (bp)**
Veuro-F	GGCACACTTTACAGTAATTG	148
Veuro-R	ACAAATCACAGCTTGTGTAAG	
Veuro-probe	/6-FAM/ACA CTT AGG /ZEN/GCA TTC GGT GAC AGG G/IABkFQ/	
Standard DNA	ATGGCACACTTTACAGTAATTGGGCTAGGACGATTTGGCATAGCCGCAAGTTTAGAGCTCATACACTTAGGGCATTCGGTGACAGGGGTAG ATCGAGAGTCGAAAATAGTGAGTAAGTATGTTGAAGCGCTTACACAAGCTGTGATTTGTGA	152

Table S2. Statistical results of data processing.

Clean reads are the raw sequences filtered the low quality, that is Clean reads which are finally used for subsequent analysis; Clean data is the number of bases in the final Clean reads; AvgLen is the average length of Clean reads; Q20 and Q30 are the percentage of bases that have quality values in Clean reads greater than 20 (sequencing error rate less than 1%) and 30 (sequencing error rate less than 0.1%); GC (%) represents the content of GC bases in Clean reads; Effective (%) represents the percentage of the number of Clean reads to the number of Raw PE.

Sample	Clean_reads	Base	AvgLen
P1	22614	32744766	1447
P2	23200	33540529	1445
P3	21201	30475226	1437
P4	22609	32610772	1442
P5	21826	31743489	1454
P6	21293	30800763	1446
P7	22261	32396513	1455
P8	23694	34397135	1451
P9	23636	34352986	1453
P10	21703	30991847	1427
P11	22450	32487463	1447
NP0.5	23475	34207232	1457
NP1	21259	30687223	1443
NP1.5	21939	32035426	1460
NP2	23534	34283665	1456
NP2.5	22916	33511970	1462
P+SC0.5	22299	32365409	1451
P+SC1	23423	34352670	1466
P+SC1.5	23407	33795423	1443
P+SC2	22663	32854556	1449
P+SC2.5	21862	31799107	1454
P+SC5.5	23903	34412263	1439

Table S3. Collapsed ASVs by Eztaxon.

Collapsed ASV	Species	Original ASVs
ASVB1	*Streptococcus thermophilus/S. salivarius/S. vestibularis* (*Streptococcus*)	ASV33, ASV36, ASV40, ASV95, ASV106, ASV143, ASV139, ASV133
ASVB2	*Vibrio gigantis/V. celticus/V. coralliirubri/V. artabrorum/V. bathopelagicus/V. crassostreae/V. pomeroyi (Vibrio)*	ASV44, ASV56, ASV81, ASV76, ASV124, ASV149, ASV147
ASVB3	*Psychrilyobacter atlanticus/P. piezotolerans (Psychrilyobacter)*	ASV18, ASV22, ASV49, ASV53, ASV52, ASV105, ASV104
ASVB4	*Vibrio europaeus*	ASV0, ASV2, ASV7, ASV8, ASV9
ASVB5	FM995181_s (Bacteria)	ASV5, ASV14, ASV17, ASV21, ASV25
ASVB6	*Escherichia fergusonii/Shigella flexneri/E. coli/S. sonnei (Escherichia)*	ASV89, ASV80, ASV68, ASV134
ASVB7	*Acinetobacter lwoffii/Prolinoborus fasciculus/A. pecorum (Acinetobacter)*	ASV3, ASV11, ASV16, ASV6
ASVB8	*Poseidonibacter lekithochrous*	ASV15, ASV27, ASV42
ASVB9	*Cutibacterium acnes*	ASV39, ASV47, ASV120
ASVB10	*Pseudoalteromonas distincta/P. elyakovii/P. atlantica/P. paragorgicola/P. arctica/P. agarivorans/P. espejiana/P. carrageenovora/P. undina/P. nigrifaciens/P. hodoensis (Pseudoalteromonas)*	ASV127, ASV115, ASV82
ASVB11	*Anaplasmataceae*	ASV24, ASV28
ASVB12	*Sphingobium limneticum*	ASV41, ASV45
ASVB13	*Brevundimonas huaxiensis/B. vesicularis/B. nasdae/B. fontaquae/B. intermedia/B. huaxiensis (Brevundimonas)*	ASV20, ASV26
ASVB14	*Algibacter*	ASV135, ASV145
ASVB15	CP014505_s (*Caballeronia*)	ASV34, ASV117

2 Supplementary Figures

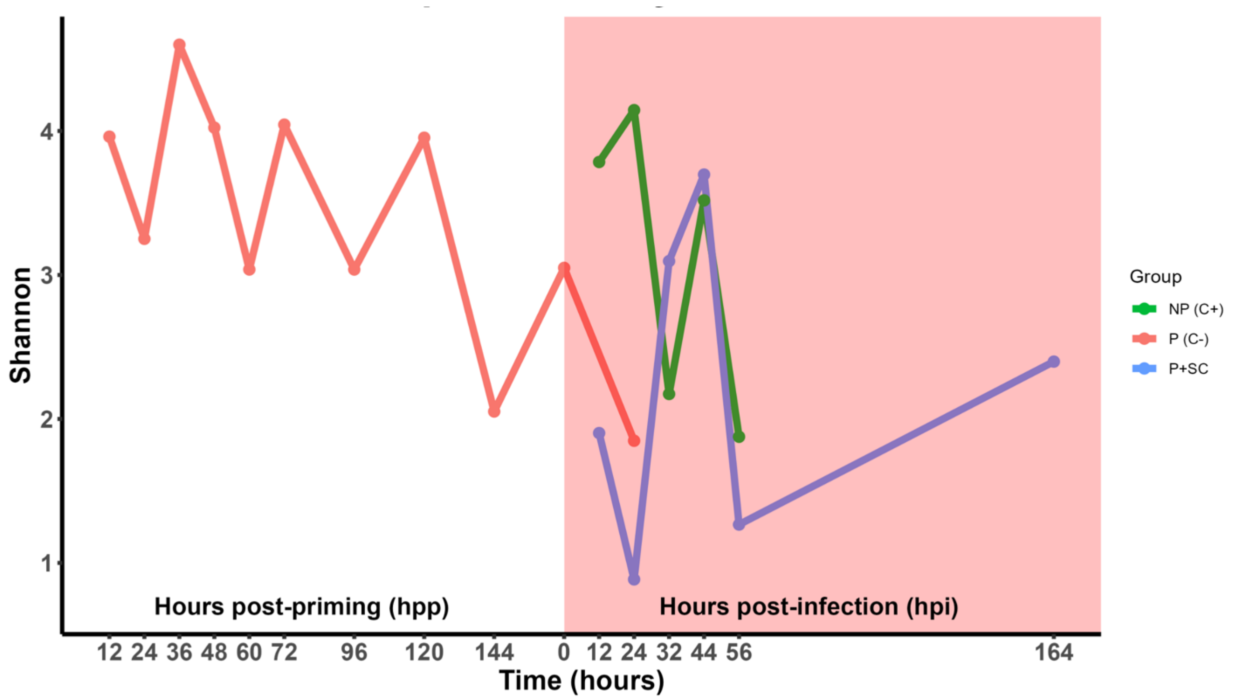

Supplementary Figure S1. Alpha diversity analyses of the Manila clam microbiota.

Apha diversity (Shannon's index) across experimental groups during the priming phase and the second challenge. Variations in diversity are shown for primed clams (P, red line), primed and subsequently challenged clams (P+SC, blue line), and non-primed clams (NP, green line) (the pink shaded area indicates the time points corresponding to the second challenge).

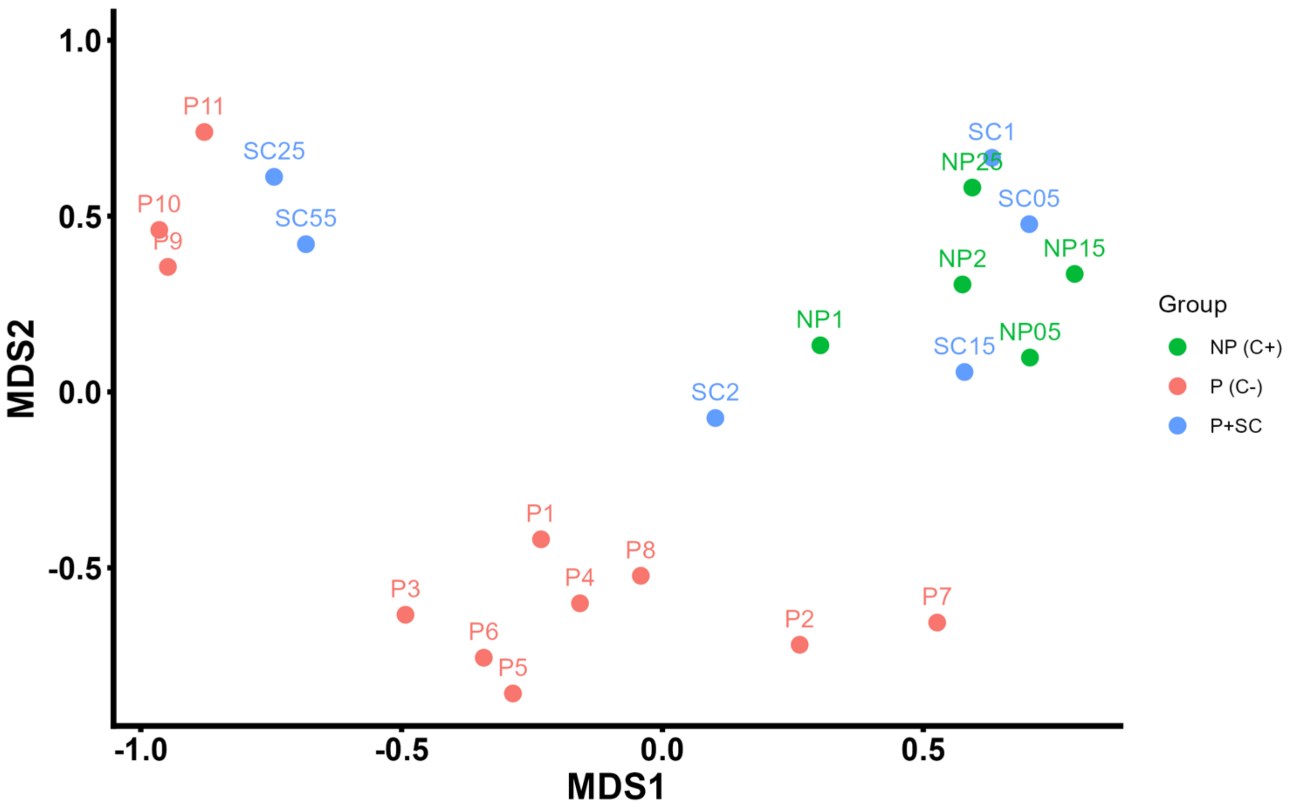

Supplementary Figure S2. Beta diversity analyses of the Manila clam microbiota based on the Jaccard distance.

Beta diversity visualized by multidimensional scaling (MDS) based on the calculation of the Jaccard Distance. Pooled Samples collected during the priming phase (P1–P11, red) and the second challenge (P+SC, blue and NP, green) are shown.

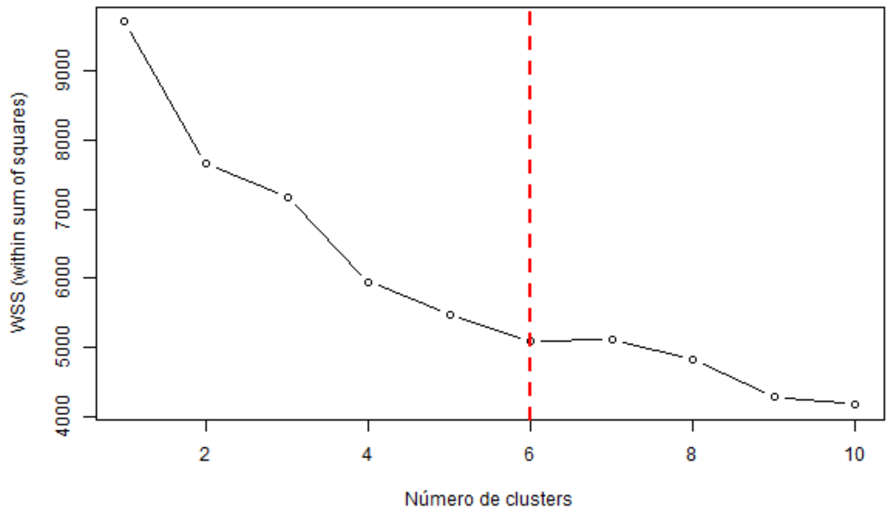

Supplementary Figure S3. Determination of the optimal number of clusters using the Elbow Method. The within-cluster sum of squares (WSS) was calculated for different numbers of clusters (k). The inflection point at $k = 6$ indicates the optimal number of clusters used for subsequent analyses.

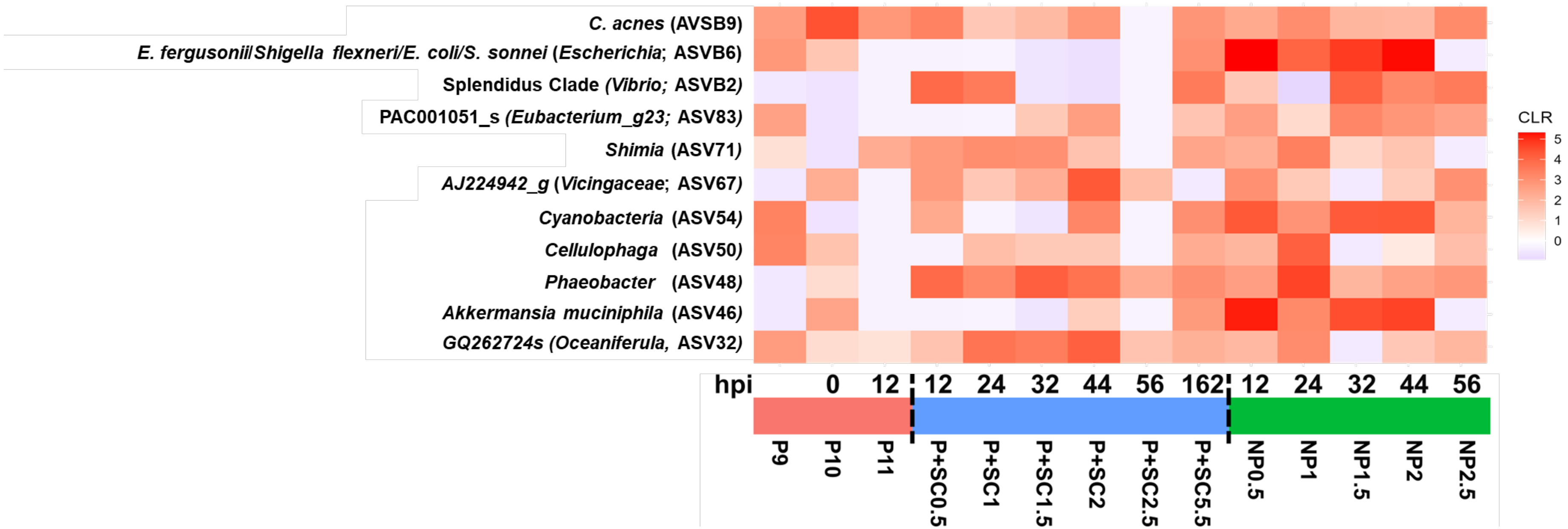

Supplementary Figure S4. Cluster 4SC.

Characteristics are showed on Fig. 5.

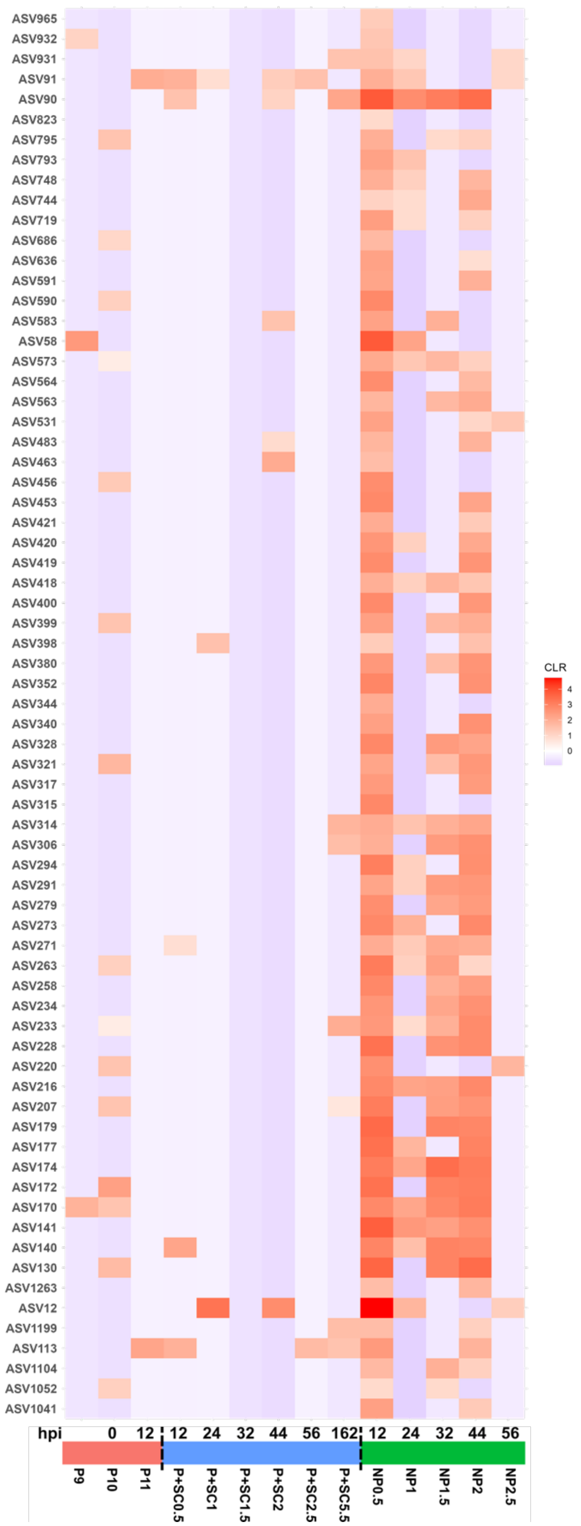

Supplementary Figure S5. Cluster 3SC.

Characteristics are showed on Fig. 5.

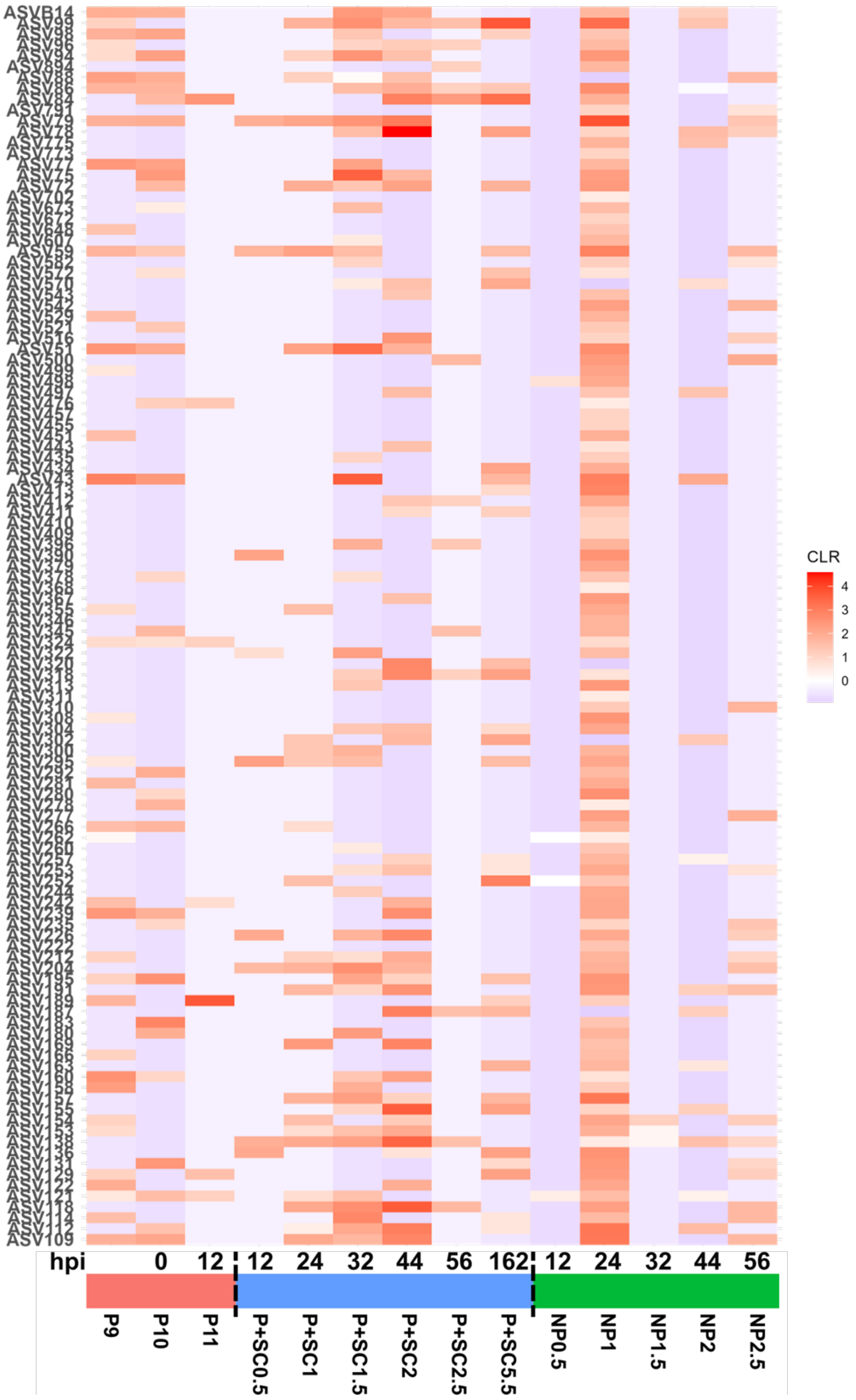

Supplementary Figure S6. Cluster 6SC.

Characteristics are showed on Fig. 5

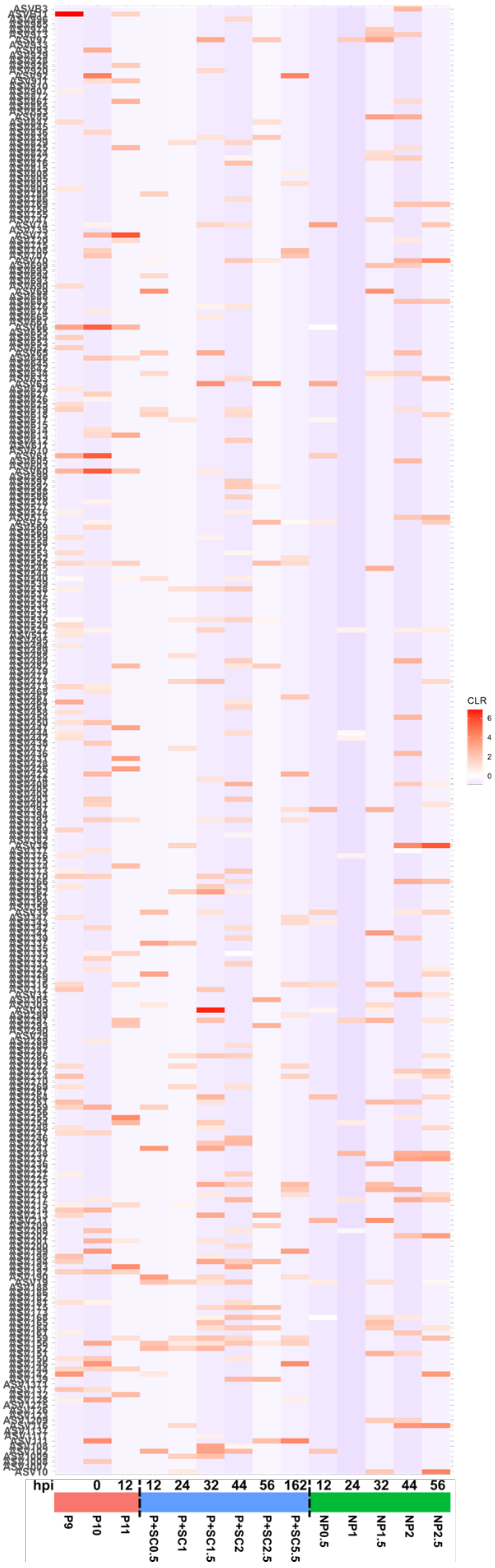

Supplementary Figure S7. Cluster 2SC.

Characteristics are showed on Fig. 5
